# Automatic ganglion cell detection for improving the efficiency and accuracy of hirschprung disease diagnosis

**DOI:** 10.1038/s41598-021-82869-y

**Published:** 2021-02-08

**Authors:** Ariel Greenberg, Asaf Aizic, Asia Zubkov, Sarah Borsekofsky, Rami R. Hagege, Dov Hershkovitz

**Affiliations:** 1 Institute of Pathology, Tel-Aviv Sourasky Medical Center, 6 Weizmann Street, 6423906 Tel Aviv, Israel; 2 Sackler Faculty of Medicine, Tel-Aviv University, Tel Aviv, Israel

**Keywords:** Gastroenterology, Computational science

## Abstract

Histopathologic diagnosis of Hirschsprung's disease (HSCR) is time consuming and requires expertise. The use of artificial intelligence (AI) in digital pathology is actively researched and may improve the diagnosis of HSCR. The purpose of this research was to develop an algorithm capable of identifying ganglion cells in digital pathology slides and implement it as an assisting tool for the pathologist in the diagnosis of HSCR. Ninety five digital pathology slides were used for the construction and training of the algorithm. Fifty cases suspected for HSCR (727 slides) were used as a validation cohort. Image sets suspected to contain ganglion cells were chosen by the algorithm and then reviewed and scored by five pathologists, one HSCR expert and 4 non-experts. The algorithm was able to identify ganglion cells with 96% sensitivity and 99% specificity (in normal colon) as well as to correctly identify a case previously misdiagnosed as non-HSCR. The expert was able to achieve perfectly accurate diagnoses based solely on the images suggested by the algorithm, with over 95% time saved. Non-experts would require expert consultation in 20–58% of the cases to achieve similar results. The use of AI in the diagnosis of HSCR can greatly reduce the time and effort required for diagnosis and improve accuracy.

## Introduction

Hirschsprung's disease (HSCR) is the most common cause of functional bowel obstruction in children with a world-wide incidence ranging between 1:5000 and 1:10,000 live births^1^. The disease is characterized by a complete lack of normal ganglion cells within the Meissner and Auerbach plexuses of the colonic wall (aganglionosis), beginning at the internal anal sphincter and extending proximally. Involvement of the recto-sigma is most common (70–85%), yet the disease may also encompass the entire colon and rarely, even the distal small bowel^2–4^.

Treatment of HSCR involves resection of the affected segment. Histological assessment is used to determine the required length of resection both pre and intra-operatively via frozen sections. In addition, post-operative assessment is required to ensure that the affected segment had been excised in its entirety^5–7^.

In HSCR the pathologist has to detect an extremely rare event of a single ganglion cell within dozens of slides. This diagnostic process is time and resource consuming, requiring multiple sections and often also immunohistochemical stains for acetylcholine esterase and calretinin^8^. Despite these measures, inconclusive biopsy results are not uncommon (range 11–38%) and inter-observer variability among pathologists may exceed 20%^9^. Errors in diagnosis have major implications. Failure to identify ganglion cells would result in a false-positive diagnosis and unwarranted surgery with loss of healthy bowel. On the other hand, a false-negative results would lead to insufficient surgery, possible persistence of symptoms and need for additional surgery^10^. Therefore, there is a need for more accurate and cost effective diagnostic tools.

Artificial intelligence (AI) and Machine learning are emerging technologies that can be used to create algorithms capable of decision making. These technologies are based on statistical methods which mimic cognitive processes and therefore enable active and continuous "learning" and improvements in the performance of the algorithm, as more raw data is provided^11^. Clinical applications include digital image analysis in various modalities in radiology^12–14^, in some instances even showing superiority over a human observer^15^.

Applications in pathology rely on recent developments and the shift to digital pathology. Machine learning tools have been used to address different clinical questions, such as histological grading^11,16–18^, determination of tumor cellularity^19^, tumor classification^20^, automated diagnosis^21–23^, as well as applications in molecular pathology^24^. Superiority over a human observer has been demonstrated as well, for instance in the classification of melanoma^25^. However, one significant limitation to the generation of clinically useful diagnostic algorithms is that the process requires collection and annotation of very large datasets. One such notable example is the recent study by Campanella et al*.*^26^ which was based on a dataset from 15,187 patients and 44,732 whole slide images. In this study the authors further concluded that as a general rule, at least 10,000 whole slide images would be required to achieve satisfactory performance with similar deep learning algorithms.

Hirschsprung's disease is relatively rare, posing a significant challenge for diagnostic algorithm development since a data set of 10,000 slides or more is simply not feasible for any given institute and would prove quite challenging even with several hospitals working in collaboration. As a demonstrative example, the total number of live births in the United States for 2018 was roughly 3.8 million^27^. Assuming an average HSCR incidence of roughly 1:8000, less than 500 cases would be expected per year. Meaning, achieving a dataset as large as the one used by Campanella et al*.* (over 15,000 cases) would require the gathering of all HSCR cases in the entire US for over 30 years. These inherent difficulties may also explain why the application of AI and machine learning in Hirschsprung's disease remains a relatively unexplored territory.

Deep learning methodology is infeasible in terms of collecting and annotating sufficient amount of data as there are no sufficient cases of HSCR. In addition, each slide contains more than ${10}^{7}$ candidates for ganglion cells, meaning each case may contain more than $0.5*{10}^{9}$ candidates. Therefore, a false alarm rate of 1% would create millions of false alarms for each case which will make the solution noisy and not effective at all. Therefore, in this study, we created and tested a tailored AI method that imitates the diagnostic procedure performed by a pathologist. Deep Learning mechanisms are used as part of the solution in a limited fashion. We called the method developed in this study Hierarchical Contextual Analysis (HCA). HCA is a method to overcome the challenge of limited datasets in HSCR in order to construct an algorithm capable of identifying ganglion cells in H&E stained slides and to assess its possible applications in the diagnosis of Hirschsprung's disease.

The diagnosis of HSCR must be performed by a trained pathologist. Our goal is to detect the often rare occurrence of ganglion cells and to present them in a graded fashion, thus assisting the pathologist in reaching the correct diagnosis (positive or negative for ganglion cells) while reducing workload and saving time.

## Results

**Figure 1:**
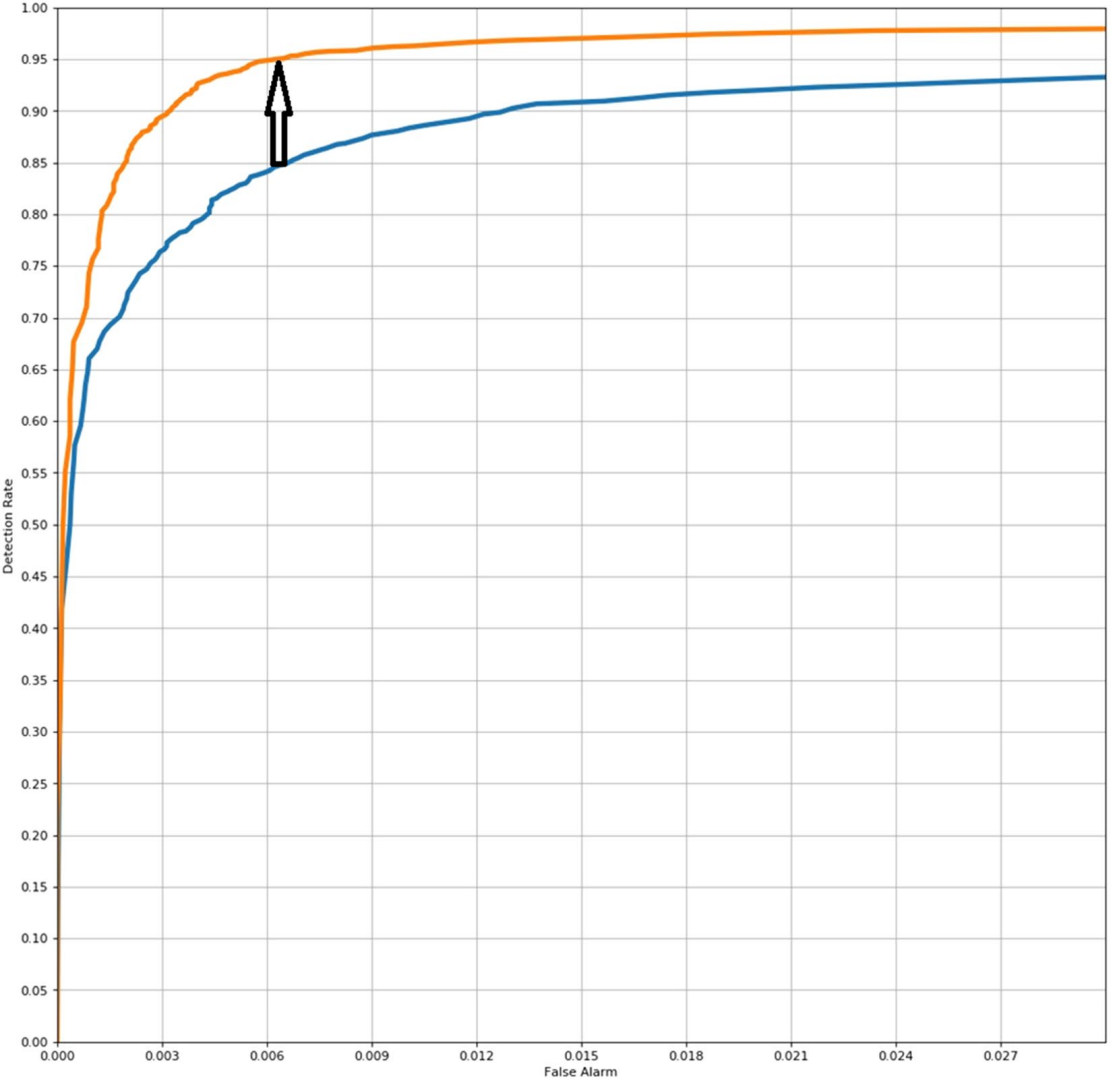
A ROC curves depicting the improvement in the performance of the algorithm after a single iteration. The algorithm's detection rate is presented in relation to the frequency of a "False alarm" (a false positive result). The blue curve predates the orange curve and is based on less data (one iteration apart). Improvement in performance (for the orange curve) is evident as a higher detection rate for each respective frequency of "False alarm" and is further stressed by the black arrow between the two curves.

In the normal colon validation cohort, the algorithm was able to identify ganglion cells with 96% sensitivity and 99% specificity (calculated on a cell-by-cell basis, in multiple areas of interest containing thousands of ganglion cells, Fig. 1).

In the validation cohort of cases suspected for HSCR: the algorithm selected 12 areas suspected for containing ganglion cells, which represent less than 0.01% of the total tissue area. The averages of the three highest scores for each set of images viewed by each observer were plotted and compared (Supplementary Fig. S1).

The algorithm successfully included at least one ganglion cell in its chosen image set (up to 12 images), for all of the cases which indeed contained ganglion cells. Therefore, the system showed a sensitivity of 100% for detecting ganglion cells with an estimated time requirement of less than 5% of that of a full analysis by a pathologist. Furthermore, for the experienced pathologist (an expert with experience with HSCR diagnosis and no previous experience with the proposed algorithm) using this algorithm yielded perfectly accurate diagnoses for all cases with an estimated time saved of at least 95% (rough estimation), when compared to conventional methods. With one exception, a "positive" diagnosis (non-HSCR) could be reached based solely on the first 3 image sets (highest AI score). Thus, the time for diagnosis per case, could potentially be reduced from around 30–60 min to mere seconds (based on the senior expert's previous experience).

For non-experts, criteria were set to determine whether a particular case could be resolved by the pathologist with use of the AI alone, or if an expert consult was required. The criteria used are detailed under "Methods". Expert consultation (microscopic examination of AI images) was considered necessary for any case classified as in "Doubt". Under these criteria, non-expert pathologists could reach 100% accuracy using only the images selected by the algorithm while consulting an expert in 20–58% of cases. Importantly, for each consultation case the expert would only need to go through the first 3 images suggested by the algorithm to reach a full diagnosis (ganglion cells present or absent). Overall, we estimate a 95% time reduction in cases suspected for HSCR diagnosis (for an expert). The time reduction may be even greater for non-experts, who are in a greater need of assistance.

Inadequate sampling was found in five cases (anal location, superficial biopsy). All inadequate cases contained no ganglion cells and were properly classified as "negative" for ganglion cells under the set criteria.

In two cases, both the pathologists and AI were in agreement among themselves, yet in disagreement with the hospital records. In both cases, the pathologists and the algorithm identified ganglion cells, whereas the pathology reports in the hospital records were negative for ganglion cells. The cases were reviewed thoroughly by the clinical team. One case represented a technical error, due to the inclusion of the proximal margin (which contained ganglion cells) in the analysis by the algorithm and later the pathologists (Case 49). No ganglion cells were identified outside of the proximal surgical margin, by either the pathologists or the AI. The other discordant case, was found to indeed represent a non-HSCR case and therefore had previously been misdiagnosed (Case 21). Analysis of the clinical records showed that the case had been revised and ganglion cells were identified in the revision. Furthermore, the patient did not undergo further surgery and his clinical follow-up was uneventful supporting the presence of ganglion cells in the tissue. Thus, the algorithm had successfully identified a previously (originally) misclassified case. Of note, one additional case (Case 50) was inconclusive due to insufficient biopsy. Cases 49 and 50, were therefore excluded from the following analysis.

**Figure 2:**
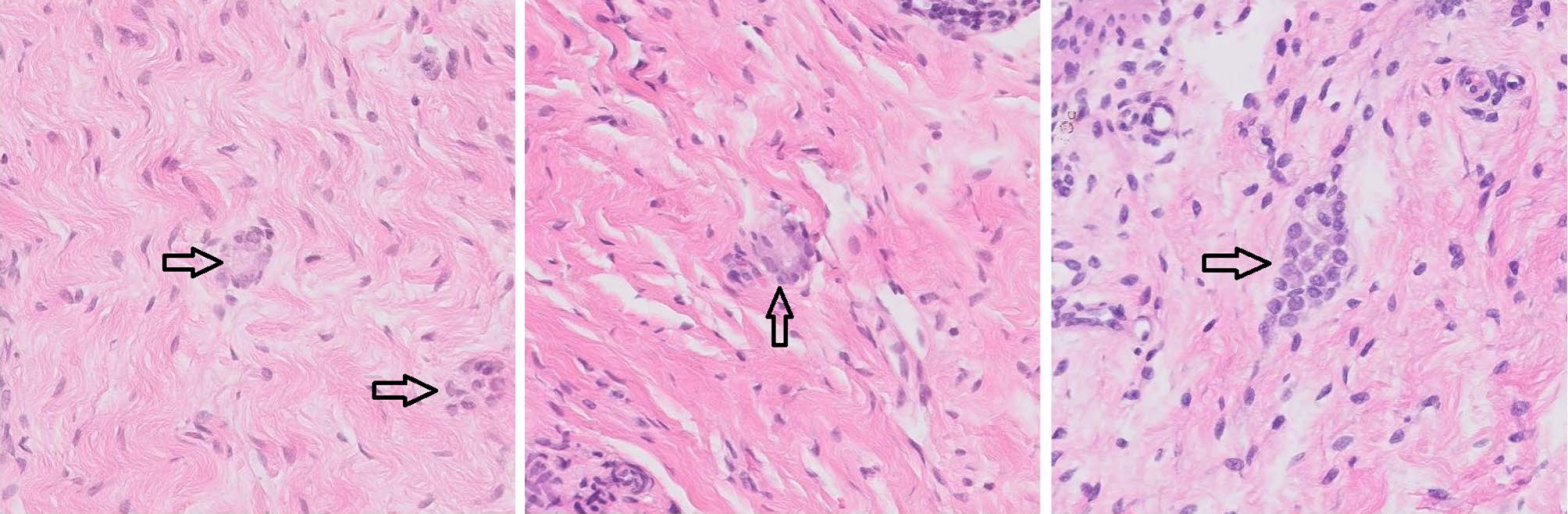
Immature ganglion cells. Examples from non-HSCR cases without evidence of mature ganglion cells. Clusters of immature ganglion cells are marked by black arrows. Note the smaller nuclei, inconspicuous nucleoli and scant intensely stained cytoplasm.

Among the non-expert pathologists, pathologist 2 (senior) required consultation in 18 cases, pathologist 3 (resident, participated in the algorithm's training) required consultation in 10 cases, pathologist 4 (resident) required consultation in 29 cases and pathologist 5 (young senior) required consultation in 18 cases. When examining consultations across all pathologists: in 9 cases (18.75%) all four of the non-expert pathologists required expert consultation to reach a diagnosis as opposed to three of the pathologists in 4 cases (10.42%), two in 9 cases (18.75%) and one in 6 cases (12.5%). Interestingly, analysis of the cases that required consultation by more non-expert pathologists showed that many of these cases contained immature ganglion cells (Fig. 2) that are indeed more challenging histologically.

Additionally, two of the pathologists have made errors in their diagnosis which were also not referred to an expert. Pathologist 5 had mistakenly diagnosed one "Negative" case as "Positive", while pathologist 3 had made one error of the same type and additionally mistakenly diagnosed a case a "Positive" case as negative.

## Discussion

The focused nature of diagnosis or exclusion of HSCR (presence or absence of ganglion cells), supposedly renders it a good candidate for automatization through AI and deep learning methods. However, several challenges must be faced in order to do so. First, the relative rarity of the disease limits the amount of data available for training an algorithm. Conventional deep learning methods would have required thousands of slides to achieve satisfactory results^26^. For this reason, we opted for the use of HCA. HCA exploits various insights provided by expert pathologists regarding the immediate context in which the observations were found and work simultaneously in different hierarchical levels to imitate those insights. Imitating those insights boosts the performance of the system considerably despite the presence of a very small database and help the system generalize in a very efficient manner. As a demonstrative example, the input by a pathologist that "ganglion cells are not present in the intestinal epithelial layer" is equivalent to a significant amount of tagged data.

Secondly, identification of ganglion cells in cases suspected for HSCR is a diagnostic challenge, both for a pathologist and perhaps even more so for an AI.

A specimen may contain few if any ganglion cells. To properly search for them, the International workspace group of the London classification recommends the initial assessment of 50–75 H&E slides for each biopsy^28^. A single sample may yield as many as one million Full HD screens (in full resolution), each image contained 100,000–200,000 pixels, while a ganglion cell is roughly 100 × 100 pixels in size. Meaning each image contained about 10,000 × 20,000 possible candidates, or 200 million candidates. Each case included a few dozen images with the total number of possible candidates reaching tens of billions. Furthermore, a single ganglion cell may be enough to rule out the diagnosis, and it may be found in any of the sections.

Consequently, few studies had attempted the use of AI in the diagnosis of HSCR.

Of note, is a study conducted by Schiling et al., which assessed the application of AI in the diagnosis of HSCR in histological slides stained for calretinin and MAP2^29^. The study was performed using 93 tissue blocks from 31 specimens of 27 patients. The reported sensitivity and specificity were 87.5% and 80% (respectively) in a training set and 95% and 90.4% in a development set.

Our study differs in aims, scope and methods. First, to the best of our knowledge, our study is the first attempt at identifying ganglion cells using AI on H&E stained slides, without the use of immunohistochemistry. Second, though still modest, our dataset was larger comprising hundreds (over 800) of slides. Lastly, Schiling et al. used conventional machine learning methods, while our study employed alternative methods. The algorithm constructed in this study, was able to exclude HSCR in 100% of the cases which contained ganglion cells (in any number).

The algorithms true strength however, lies not in its individual performance, but in its application as an assisting tool for the pathologists in their evaluation of HSCR cases.

In the present study, an accurate diagnosis or exclusion of HSCR could be made by an expert pathologist within minutes and in some cases even seconds, as opposed to hours, by traditional methods. Use of the algorithm may lessen the need for immunohistochemical stains and thus has the potential to save costs both direct and indirect (through greater efficiency in the pathologist's time). More importantly, the algorithm's high sensitivity may aid the pathologist in reaching the correct diagnosis, ensuring that no ganglion cells are missed (thus, incorrectly diagnosing HSCR).

Upon review of the literature regarding the diagnosis of HSCR, the reported specificity is almost uniformly high (closer to 100%) and therefore false-positive results are rare. However the rate of false-negative results varies widely between 0 and 40%^30^.

Immunohistochemistry is often employed to improve the diagnostic accuracy. Commonly used stains include Acetylcholine esterase (AchE), Calretinin, S-100 and MAP-2 and more, used alone or in combination (panels)^31–35^. Serafini et al. compared the diagnostic accuracy of H&E alone or Calretinin staining versus AchE staining as a gold standard. H&E alone had a 90% concordance with AchE staining and demonstrated a higher diagnostic accuracy (96%) and specificity (78%) yet a lower sensitivity (70%) when compared to Calretinin staining (84%, 70% and 96%, respectively)^36^. Other studies reported higher accuracy for Calretinin, as an example, Jeong et al., reported a sensitivity of 100%, 93.5% and 100%, a specificity of 34.4%, 100% and 85.2% and an accuracy of 57.9%, 97.8% and 90.5% for H&E, AchE and Calertinin, respectively^37^. Kapur et al*.* stained for the choline transporter as a substitute for AchE staining and demonstrated a total error rate of about 20%^9^. In our study, an experienced pathologist directed by the algorithm was able to achieve 100% detection rate (sensitivity) and 0% false alarms (100% specificity), without the need for any additional aids including the full slides or additional stains. Non-expert pathologists could still reach 100% while requiring expert consultation in 20–58% of cases. Therefore, use of the algorithm may simplify the diagnosis and allow even less experienced pathologists to perform adequately. This is especially important for smaller or secluded institutions which may not receive enough HSCR cases to allow building of expertise. Furthermore, most consultations could be concluded based solely on the 3 images with the highest scores, allowing for shorter, more accurate and less expensive consultations.

Inter-observer variability was significant (wide range of scores and need for consultation). However, the algorithms main goal is to aid the pathologists in reaching the correct diagnosis. This goal has been achieved in all cases, under the set criteria and despite any variability present.

Two cases, demonstrated discordance between the algorithm (and later the pathologists) and the pathological ground truth as stated in the hospital records. One of these two cases was classified as HSCR yet in fact harbored a few ganglion cells, which the algorithm was able to correctly identify. The implications of such a misdiagnosis may be additional surgery and loss of a greater length of bowel than clinically necessary. In this particular case, the patient was luckily managed without need for additional surgery. Even so, use of the algorithm might have been able to prevent this mistake and similar mistakes, potentially preventing unnecessary bowel resection, as well as additional, unindicated surgery with its associated risks and complications. In the second case, the proximal margin, likely representing the transition zone was included by mistake. As this margin contained true ganglion cells, which were identified by both the algorithm and the pathologists, the case was mistakenly marked as non-HSCR. As with the other cases, in this specific sample the algorithm had worked as intended, and was able to identify the few present ganglion cells in a single slide out of dozens.

An additional case proved inconclusive even for an expert, who commented that in a real time clinical setting, an additional biopsy would have been recommended. This approach is not uncommon in clinical practice. In this particular case, the patient's follow-up is unavailable.

The relatively high amount of referrals (20–58%) recommended in this study should be inspected in the context of the decision criteria set. The criteria were intentionally set in a way which would likely cause over-referral, yet would minimize diagnostic errors. Indeed, nearly all of the mistakes made by the non-expert pathologists were revealed and corrected.

Certain cases proved more challenging for the non-expert pathologists, resulting in most or all of them requiring expert consultation. When examined, cases which were positive for ganglion cells (non-HSCR) yet confusing for the non-experts contained immature ganglion cells. Immature ganglion cells are morphologically different from the more familiar mature ganglion cells, and are therefore more likely to be misclassified by a non-expert pathologist. They have smaller nuclei, inconspicuous nucleoli, as well as a scant, yet more intensely stained cytoplasm. Their appearance may mimic plasma cells or lymphocytes. Helpful clues are their tendency to appear in groups, proximity to blood vessels, as well as persistence in serial secions^38^.

**Figure 3:**
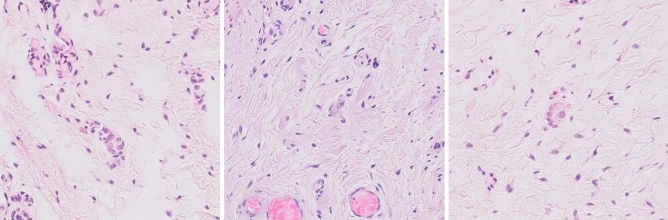
Examples of non-specific findings, mistaken for ganglion cells by non-expert pathologists. Without proper context, these findings may mimic immature ganglion cells. However, many of these findings were in fact within the mucosa or serosa, bowel layers normally devoid of ganglion cells, thus ruling out any "ganglion cells" seen within them.

Cases which were negative for ganglion cells (HSCR) yet had high referral rates, had mostly non-specific and out of context findings. The algorithm searches for the best ganglion cell candidates. However, in a slide devoid of ganglion cells, the "best" candidates are still "bad" candidates which are unlikely to represent true ganglion cells, yet are the closest entities to be found (Fig. 3). As mentioned, the pathologist is aware of the optional presence of immature ganglion cells yet lacks the experience to properly identify them, which may result in the interpretation of non-specific findings as possible immature ganglion cells. Furthermore, in the original version of the algorithm, the images were presented to each pathologist without additional context (immediate surroundings aside). Ganglion cells are only found within the submucosa or muscularis propria. Any cell or finding within any other layer, no matter how superficially similar is highly unlikely to represent a true ganglion cell. However, without this context, some findings can mimic ganglion cells, particularly immature ones, enough to cause the non-expert great unease. Such cases are much more likely to be referred for an expert consultation. Of note, future applications of the algorithm will include the origin of each image within its respective slide in order to provide better context.

Using the decision algorithm, the pathologist might still make a false positive diagnosis which would not sent to consultation, if a score 5 is given inaccurately for 2 ganglion cell candidates. Further improvements to the algorithm as well as training of the pathologists in use of the algorithm will likely minimize future mistakes of this nature.

This study had several limitations which merit mention. As stated, the available dataset was limited and significantly smaller than that of similar studies on the use of AI in pathology^17,18,21,25,26,29^. Large data sets are considered necessary in order to properly represent the wide variability present in clinical samples^26^. Smaller data-sets therefore suffer both from a statistical standpoint and from excessive uniformity. Our use of HCA somewhat circumvents this problem. Nevertheless, additional data, including data generated by other institutions and other slide scanning platforms would allow for further validation which could improve upon the algorithm^39,40^.

The main limitation of the HCA method itself lies in is its reliance on input and feedback from a pathologist. Meanwhile, conventional deep learning methods may operate mostly independently, at the cost of requiring far larger data sets. In the case of HSCR large datasets are not available making HCA the only real option for effective algorithmic solution.

In conclusion, the algorithm constructed in this study is an excellent addition to the ever growing "toolbox" of AI and digital solutions that are rapidly being made available to pathologists worldwide. Additionally, HCA, the algorithmic approach taken in this study, could be further applied to other diagnostic scenarios to achieve satisfactory performance even with a limited data set**.** The same process would also serve to better validate the HCA approach by providing additional data from additional experts, institutes, clinical settings and scanning modalities. This may aid the development of diagnostic and assisting tools using AI and deep learning.

## Materials and methods

All methods were performed in accordance with the relevant guidelines and regulations.

### Ethics statement

All data used in this study was derived from digital pathology slides identified only by a sample number and no other identifying details. The study was approved and informed consent was waived by the local ethics committee at Tel-Aviv Sourasky medical center. Approval number: 0660-16-TLV.

### Algorithmic approach

*Deep learning* relies on extensive amounts of data which is not available in the case on HSCR. Additionally, the performance that can be achieved by applying these methods even given a sufficient amount of data would not lead to high enough performance. Therefore, the only research direction we could use, in order to create and test an AI method that can assist the pathologist in the diagnosis of Hirschsprung disease, was to develop an alternative method which we call the hierarchical contextual analysis (HCA).

HCA is based on two observations regarding the fundamental ways in which pathologists assess and determine whether what they see is indeed a ganglion cell. We identified two aspects to the pathologist's diagnostic process, as follows:

*Hierarchical analysis* When observing a given region within a tissue slide the pathologist has very clear knowledge about the tissue type, the location within the tissue and the geometrical location and orientation relative to other detected entities in the slide. For example, ganglion cells are not found in the surface epithelium, and any similar event found in this location, must be interpreted as "Negative". This method eliminates a massive amount of false alarms and makes the analysis much more efficient.

*Contextual analysis* In the moment a candidate region is detected as a potential ganglion cell, it is positioned and compared to its immediate surroundings, as well as to other, candidate ganglion cells. For example, a ganglion cell may appear in clusters accompanied by other types of cells, such as schwann cells. This context is crucial for differentiating between different cell types which may overlap or appear partially within a given slide.

The observations made about the way in which the pathologist reviews tissue slides in order to reach high performance in an effective way were imitated in HCA. While deep learning techniques can be thought of as feedforward techniques, namely a sequence of stages from the input to the output, without any type of feedback, HCA is heavily infused with feedback loops.

The analysis of the geometric relationships between different entities in the image, as well as, the implantation and integration of these relationships through statistical techniques such as deep learning, were previously explored by us, in past works^41–43^.

Data collection for the algorithm included both an analysis of the diagnostic routine performed by the pathologist and the actual annotation of ganglion cells in digital pathology images.

The data collected on the diagnostic routine of the pathologists was collected both through extensive discussions and through direct active observation during work. Data from the images included direct annotation of ganglion cells, and suspected ganglion cells. Additionally, the part of the images surrounding candidate ganglion cells was also used for generating the algorithm (Negative labeled background).

### Clinical samples

The material used in this research was derived from formalin fixed paraffin embedded tissue. Hematoxylin and eosin stained slides were scanned using the Philips UFS scanner (Koninklijke Philips, Amsterdam, The Netherlands), at X40 magnification. The proprietary ISYNTHAX format was converted to TIFF format using the Philips IntelliSite pathology Solution program, version 3.2 and annotations were made manually (Supplementary Fig. S2), by use of the ASAP v.1.7.3 program (Geert Litjens, Nijmegen, The Netherlands). The Pathological ground truth was based on hospital records. All cases were previously reviewed by senior pathologists, most of whom with experience and expertise in HSCR diagnosis. Multiple sections and immunohistochemical stains were available when necessary at the time of the original diagnosis.

The algorithms training involved the use of 95 digital pathology slides in total: 86 slides of specimen containing adult, normal colons (obtained from colectomy specimen), and 9 additional slides of cases with a clinical suspicion of HSCR. All slides were reviewed by a single pathologist. Ganglion cells (positive samples) were marked by encircling each of them, individually. Fine tuning of the algorithm involved marking by the algorithm and feedback by the pathologist. Feedback included a statement as to whether ganglion cells were seen or not (true/false), as well as correction for false positive (excess markings) and false negative (unmarked ganglion cell) results. The initial training phase involved normal colonic biopsy slides. In 10 of these slides, manual analysis was complete and included marking all identifiable ganglion cells in the entire slide for a total of 2315 cells. The same process was performed in marked "areas of interest" in 64 additional slides. The sum of the surface of all areas of interest was less than one full slide yet contained 1476 ganglion cells in total. 10% of the database was not used in the procedure of building the algorithm and were instead used for measuring the performance of the algorithm and obtaining initial values of sensitivity and specificity in identifying ganglion cells in normal samples. Of note, the calculation of sensitivity and specificity was based on thousands of small "areas of interest" including ganglion rich areas and areas devoid of ganglion cells. The sensitivity and specificity values measure the algorithms capacity to correctly identify the individual ganglion cells in these areas on a cell-by-cell level.

The reminder of the slides as well as additional "areas of interest" in previously used slides were used for fine tuning of the algorithm (Supplementary Fig. S3). Of note, each event (ganglion cell) identified by the algorithm was identified and displayed in the context of its immediate surrounding. This was necessary, as pathologists generally work with a contextual approach in mind ("Contexual analysis") and would be justly uneasy in making a diagnosis on what appears to be a ganglion cell yet taken out of the context of its surroundings. The algorithm must therefore follow the same approach, both to imitate the diagnostic process of the pathologist, as well as to better fit the pathologist's needs when used as an assisting tool. In addition, as part of the "Hierarchical analysis" approach, "areas of interest" devoid of ganglion cells, were also marked and served as negative samples. An important and especially confounding example is the surface epithelium, which may include multiple false positive events. By providing a sufficient amount of areas of surface epithelium marked as negative, the system is able to "learn" which layers of the colonic wall are relevant and which should be ignored, in a similar fashion to a pathologist being taught where to look.

### Validation on cases with clinical suspicion of HSCR

Fifty cases (727 slides in total) with clinical suspicion HSCR were analyzed using the proposed algorithm. The specimens were obtained from suction biopsies (39, or 78%), full thickness biopsies (4, or 8%) and surgical specimen (7, or 14%). Three cases were obtained from young adults, with the remainder being pediatric patients. The age range was between 1 day and 28 years, with 39 cases being obtained from patients younger than 1 year and of them, 16 cases were obtained from patients younger than 1 month.

Adequacy of each biopsy slide (representation of the submucosa and/or muscularis propria) was not a criteria and determination of adequacy is not a feature of the algorithm. A total of five cases were considered inadequate upon review. One case included samples from the anal region, which is physiologically aganglionic. The other four were rectal biopsies which were too superficial. An inadequate sample should contain no ganglion cells. Therefore, a "negative" classification by the algorithm was considered the expected and correct function for inadequate cases.

The algorithm provided a score between 0 and 1.

Up to 36 images with the highest assigned scores had been chosen from each case and were then divided into sets of 3 images (up to 12 sets from each case). The image sets were ordered according to their AI score, from highest to lowest.

The sets were presented unmarked and unscored to be reviewed by three pathologists, one with expertise in HSCR diagnosis and two non-experts: one a senior, one a resident who provided the data for construction and training of the algorithm in the previous steps.

Each pathologist assigned a score to each set of images according to the following scale:

1. No ganglion cells seen (certain)
2. No ganglion cells (uncertain, low probability of ganglion cells)
3. Uncertain/cannot be determined
4. Ganglion cells seen (uncertain, high probability of ganglion cells)
5. Ganglion cells seen (certain)

The scores for all 3 pathologists as well as the algorithm (using its own score—probability between 0 and 1) were compared to each other and to the known status of the sample (positive or negative for ganglion cells).

The data set collected in this manner was used for the formulation of classification criteria. For the purpose of classification, when applicable, the average of the three highest AI scores for each case was used. The criteria are as follows:

For an expert pathologist, the identification of a single ganglion cell (An attributed score of 5) was sufficient to classify a case as positive for ganglion cells (non-HSCR). Otherwise (all scores < 5), the case was classified as (Negative for ganglion cells (HSCR).

For non-experts, cases were either considered "Positive", "Negative" or in "Doubt".

Cases classified as in "Doubt" were considered to require expert consultation. The criteria are as follows:

1. Positive (non-HSCR)—If the pathologist attributed a score of 5 to any 2 (or more) sets of images,
2. Negative (HSCR)—The criteria for "Positive" is not met AND the average AI score is < 0.6.
3. Doubt—The criteria for "Positive" is not met AND the average AI score is ≥ 0.6.

### Decision algorithm validation

The process described in this section was repeated with two additional pathologists, one a young senior the other a resident. Both pathologists had no prior experience with either HSCR diagnosis or the algorithm. Both received concise instructions and no prior training. No time limit was imposed. The same scoring and classification criteria were used.

## Supplementary Information

Supplementary Information 1.

Supplementary Information 2.

Supplementary Information 3.

Supplementary Information 3.
