# Supplementary figures and images for: Automatic ganglion cell detection for improving the efficiency and accuracy of hirschprung disease diagnosis
Source: Sci Rep. 2021 Feb 8;11:3306. doi: 10.1038/s41598-021-82869-y (PMC7870950; doi:10.1038/s41598-021-82869-y)

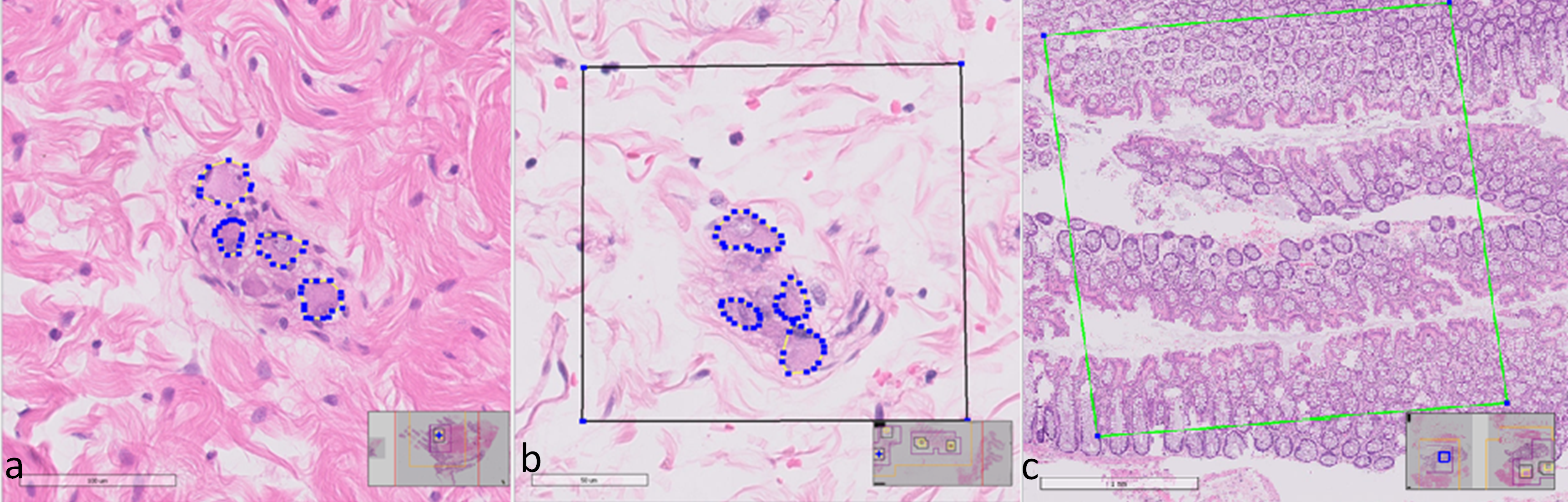

Supplement: Supplementary file 1 — Supplementary Information 1. [file 41598_2021_82869_MOESM1_ESM.tif]

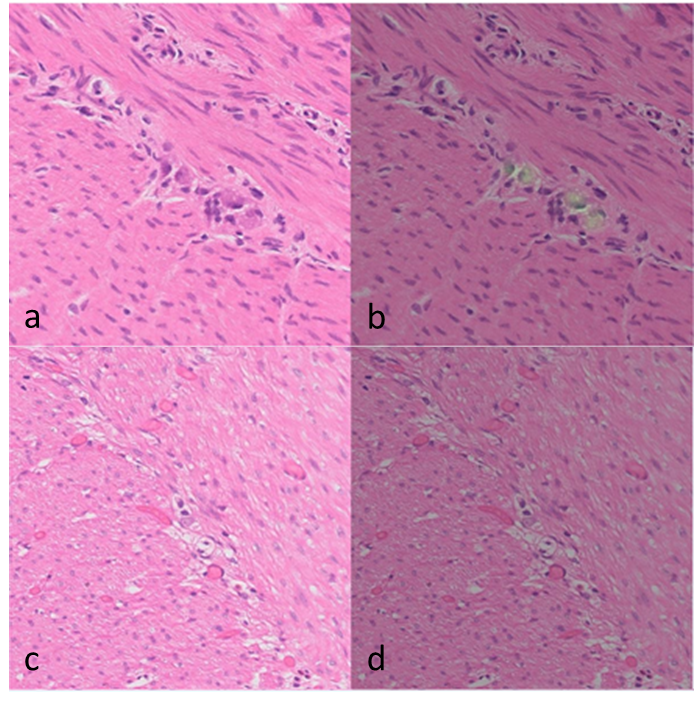

Supplement: Supplementary file 2 — Supplementary Information 2. [file 41598_2021_82869_MOESM2_ESM.tif]

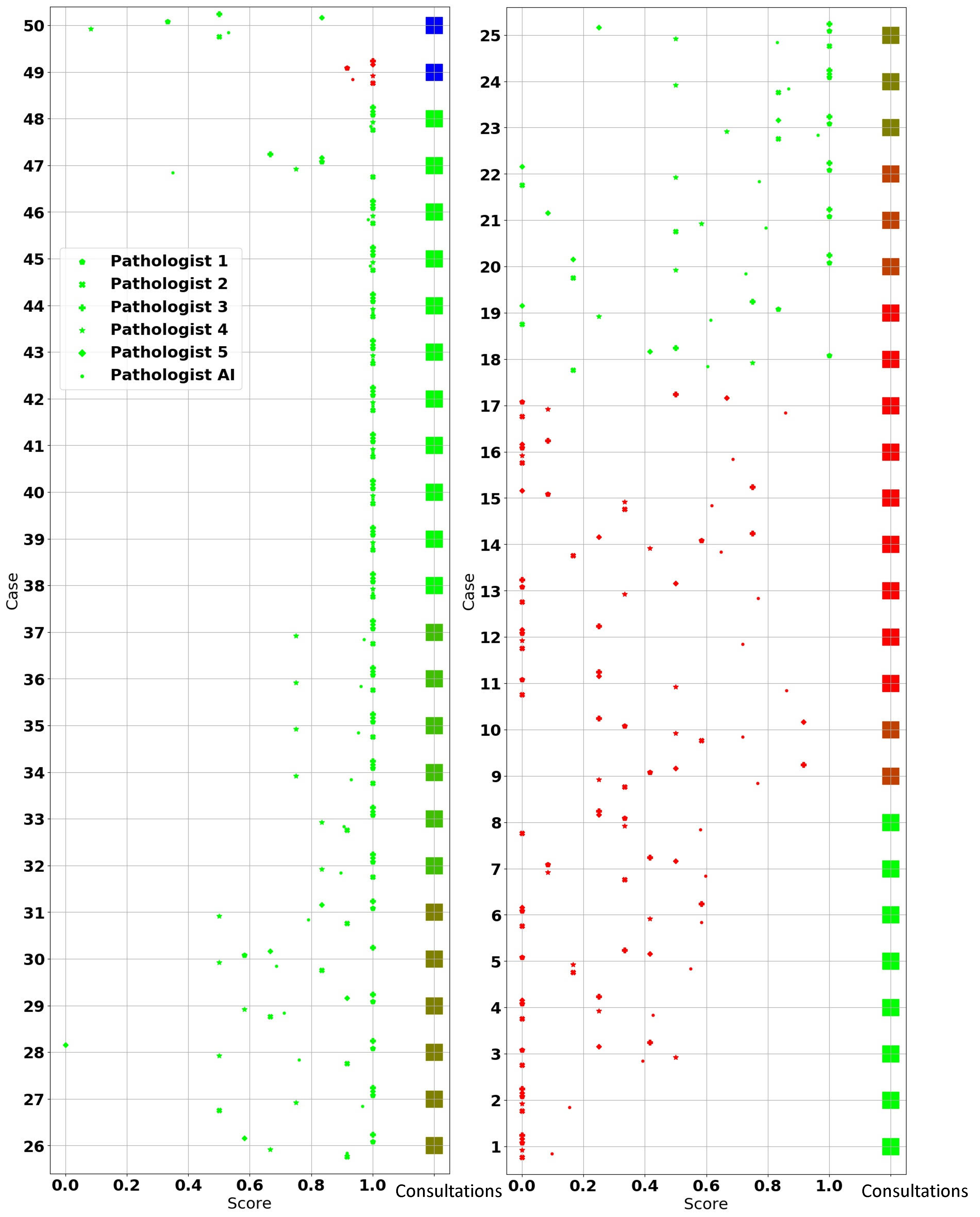

Supplement: Supplementary file 3 — Supplementary Information 3. [file 41598_2021_82869_MOESM3_ESM.tif]
