# Supplementary material for: Automatic ganglion cell detection for improving the efficiency and accuracy of hirschprung disease diagnosis
Source: Sci Rep. 2021 Feb 8;11:3306. doi: 10.1038/s41598-021-82869-y (PMC7870950; doi:10.1038/s41598-021-82869-y)
Supplement: Supplementary file 4 — Supplementary Information 3. [file 41598_2021_82869_MOESM4_ESM.docx]

**Automatic Ganglion Cell Detection for Improving the Efficiency and Accuracy of Hirschprung Disease Diagnosis**

Ariel Greenberg^1^,MD, Asaf Aizic^1^, MD, Asia Zubkov^1^,MD, Sarah Borsekofsky^1^, MD, *Rami R. Hagege^1^, Ph.D, Dov Hershkovitz^1,2^, MD, Ph.D.

*Co-equal last author

^1^Institute of pathology, Tel-Aviv Sourasky Medical center, ^2^Sackler Faculty of medicine, Tel-Aviv University, Tel-Aviv, Israel

Correspondence and reprint requests to:

Dov Hershkovitz, M.D. Ph.D.

Institute of Pathology

Tel-Aviv Sourasky Medical Center

[6 Weizmann Street, Tel Aviv, 6423906](https://www.tel-aviv.gov.il/AuctionAndCareers/DocLib2/539%20-%20%D7%A8%D7%95%D7%A4%D7%90%20%D7%9E%D7%95%D7%9E%D7%97%D7%94%20%D7%91%D7%A4%D7%A1%D7%99%D7%9B%D7%99%D7%90%D7%98%D7%A8%D7%99%D7%94.pdf)

Phone: +972-36973530

Fax: +972-36974648

e-mail: [dovh@tlvmc.gov.il](mailto:dovh@tlvmc.gov.il)

**Figure legends**

Supplementary figure 1: A diagram depicting all cases (1 through 50), each marking represents the average 3 highest scores attributed to a particular case by a single observer. The color of each marking correlates with the pathologic ground truth: Green for cases which are positive for ganglion cells, red for cases negative for ganglion cells. The scores were normalized to a 0 to 1 scale. The colored boxes at the right hand side represent the number of non-experts that required consultation for each case, ranging from light green (0), dark green (1), olive green (2), dark red-brown (3), and bright red (4). Blue colored boxes represent cases which were excluded from the analysis, as described under "results".

Supplementary figure 2: Examples of annotations made using the ASAP program to provide the initial data for the construction of the algorithm. Image "a" shows marked ganglion cells in a fully analyzed section (slide), image "b" shows marked ganglion cells in a predefined "area of interest" which was demarcated around them. Image "c" shows an "area of interest" lacking ganglion cells and serving as a negative sample.

Supplementary figure 3: Two sets of images which were used during feedback while training the algorithm. Images "a" and "b" are identical, with "a" being the original and "b' including markings of ganglion cells provided by the algorithm. Image "a" was then marked by the pathologist and submitted as feedback. Images "c" and "d" are a second set of identical images, this time lacking any ganglion cells and accordingly any markings by the algorithm.
